# Charge transport through one-dimensional Moiré crystals

**DOI:** 10.1038/srep19701

**Published:** 2016-01-20

**Authors:** Roméo Bonnet, Aurélien Lherbier, Clément Barraud, Maria Luisa Della Rocca, Philippe Lafarge, Jean-Christophe Charlier

**Affiliations:** 1 Université Paris Diderot, Sorbonne Paris Cité, Laboratoire Matériaux et Phénomènes Quantiques, UMR 7162, CNRS, 75205 Paris Cedex 13, France; 2 Université catholique de Louvain, Institute of Condensed Matter and Nanosciences, Chemin des étoiles 8, 1348 Louvain-la-Neuve, Belgium

## Abstract

Moiré superlattices were generated in two-dimensional (2D) van der Waals heterostructures and have revealed intriguing electronic structures. The appearance of mini-Dirac cones within the conduction and valence bands of graphene is one of the most striking among the new quantum features. A Moiré superstructure emerges when at least two periodic sub-structures superimpose. 2D Moiré patterns have been particularly investigated in stacked hexagonal 2D atomic lattices like twisted graphene layers and graphene deposited on hexagonal boron-nitride. In this letter, we report both experimentally and theoretically evidence of superlattices physics in transport properties of one-dimensional (1D) Moiré crystals. Rolling-up few layers of graphene to form a multiwall carbon nanotube adds boundaries conditions that can be translated into interference fringes-like Moiré patterns along the circumference of the cylinder. Such a 1D Moiré crystal exhibits a complex 1D multiple bands structure with clear and robust interband quantum transitions due to the presence of mini-Dirac points and pseudo-gaps. Our devices consist in a very large diameter (>80 nm) multiwall carbon nanotubes of high quality, electrically connected by metallic electrodes acting as charge reservoirs. Conductance measurements reveal the presence of van Hove singularities assigned to 1D Moiré superlattice effect and illustrated by electronic structure calculations.

Moiré patterns^1^ are quite fascinating phenomena resulting from the combination of two well-defined geometrical structures owing their own intrinsic properties but finally transformed as soon as they are superimposed giving rise to a new superstructure with unique properties. In art, they are quite common and are frequently used to captivate the observer since their exotic patterns may dramatically change when the observer moves around^2^. In physics, Moiré patterns have recently allowed to observe fractal figures in Hofstader’s butterfly through quantum Hall effect in bilayer graphene and in single-layer graphene deposited on hexagonal boron nitride devices^3^,^4^,^5^. Indeed, the resulting Moiré superstructure has induced an artificial enlargement (up to few nanometers) of the periodic potential seen by charge carriers whereas Bloch states are usually periodic over atomic distances^6^. 1D quantum conductors such as multiwall carbon nanotubes (MWCNTs) intrinsically present Moiré superlattices^7^,^8^. In analogy with 2D van der Waals crystals^9^,^10^, recent calculations^11^ suggested that the occurrence of Moiré superstructures in 1D systems strongly modifies the electronic properties of the device. For example, two individual semiconducting CNTs can form either an insulating, or semiconducting or even a metallic double-wall CNT (DWCNT) depending on the resulting Moiré superstructure^11^. This dramatic change in the electronic behavior can be rationalized by considering the weak but long-range interaction between the tubes which depends on the relative rotation angle^12^. Actually, Moiré pattern can serve as a probe for this specific interlayer interaction^13^. However, superlattices in MWCNTs and more specifically in DWCNTs have been experimentally investigated mainly from a structural point of view^14^,^15^. Few studies^16^,^17^ report on charge transport properties and point out the influence of the independent electronic properties of the two isolated tubes on the global electronic structure of the constituted DWCNTs. Our approach is different since we investigate by transport measurements and theoretical calculations the presence of 1D Moiré superlattices in large-diameter MWCNTs.

Our electronic devices (Fig. 1a) consist in individual MWCNTs contacted by evaporated Ni/Au electrodes (see Methods). These electrodes are evaporated on top of the MWCNT, thus allowing a contact only with the outermost shell as depicted in the inset of Fig. 1a. A cross-sectional view of a contacted MWCNT highlighting the three outermost tubes (colored) which are expected to participate to the conduction in this device is illustrated in Fig. 1b. Indeed, when the contact is deposited on the outertube, it has been demonstrated experimentally^18^ that only the outermost shells are carrying the current in the device due to a large intershell resistance. The diameter of the investigated nanotubes is also very large (≥80 nm) allowing potential Moiré patterns to develop over its surface. With such a large tube, the landscape over the surface of the outertubes is almost flat at the atomic scale recalling locally a few-layer graphene structure as depicted in Fig. 1c.

In order to develop the concept of 1D Moiré crystal, Fig. 1d–g illustrates different models of stacked honeycomb lattices. For the sake of clarity, the discussion is here restricted to two layers but Moiré patterns implying three layers will be presented later in the manuscript. Figure 1d corresponds to the simple case of AA-stacking in bilayer graphene, i.e. a perfect superposition of the two carbon lattices with no Moiré pattern. Figure 1e represents the case of a twisted graphene bilayer revealing a 2D Moiré pattern. Figure 1f–g corresponds to the case of DWCNTs. The upper (lower) images refer to the case of armchair (zigzag) lattice orientations. To explain the dilatation along the circumference (B axis) of only one lattice, the simplest argument is that the tube curvature generates a distortion equivalent to an artificial increase of the inner layer lattice (red) parameter along the CNT’s circumference. Typically, this distortion is less than 1% in the case of a 80 nm-diameter MWCNT. Following this argument, Fig. 1f–g illustrates DWCNTs with identical (Fig. 1f) and different (Fig. 1g) tubes chiralities, respectively. They both present a Moiré pattern but with different geometrical characteristics. Figure 1f highlights the 1D character of the Moiré pattern exhibiting smoothly varying and alternating stacking arrangement from AA to AB. Finally, this lateral distortion caused by closed curved surface can be combined with a twist of the walls corresponding to different tube chiralities, inducing another type of 2D Moiré pattern. In this letter, our theoretical approach is devoted to the case of shells presenting identical chirality giving rise to a fringe-like 1D Moiré pattern as depicted in Fig. 1f. Even if the chiral indices of the experimentally investigated MWCNT are not known, the calculations capture the essential physics of the experiments.

We consider large-diameter MWCNTs (≥80 nm) reduced to their only three outermost shells based on the intertube resistance argument. To ensure a global overview, two types of tube chiralities will be considered, representing the two limiting cases, namely a triple-wall CNT (TWCNT) composed of only zigzag tubes and a TWCNT composed of only armchair tubes. First, the zigzag configuration is investigated. The (1045, 0)@(1054, 0)@(1063, 0) TWCNT presents a unit cell containing 12648 carbon atoms and the three tube diameters are 81.8, 82.5, 83.2 nm, respectively. Its electronic band structure is calculated using a tight-binding model (see Methods) by solving the eigenstate problem for interacting shells. Large individual zigzag CNTs, are expected to be semiconducting with a tenuous direct band gap in Γ (i.e. for k = 0). Without any interaction between tubes, it is thus expected that the narrowest gap coming from the larger tube will dominate the low energy spectrum. However, when intertube interactions are switched on, the zigzag TWCNT is found to be metallic, as illustrated in Fig. 2a. Interestingly, a set of electronic bands form a new Dirac cone at the Fermi energy (E_F_ = 0 eV) but slightly shifted away from Γ. The crossing of these bands can be avoided, thus creating a large pseudo-gap by rotating the central tube along its axis (Fig. 2b and Fig. S1). In analogy with graphene-based 2D superlattices^9^, the presence of a long-range periodic potential in TWCNT also leads to superlattices Dirac points (DP) either at the same energy or within the hole and the electron-like bands. In addition, avoided crossing points, called pseudo-gaps (PG), can also be generated as theoretically reported in the past for small diameter MWCNTs^19^,^20^. Additional structures and singularities in the density of states (DOS) of the nanotube appear as a consequence as such band structure features. Similar evidence of those induced features in band structures due to superlattice effects has been experimentally investigated in a solid-state device (illustrated in Fig. 1a) by conductance measurements performed at 4.2K through a MWCNT and presented in Fig. 2c. First, note that the global conductance value is well below the quantum of conductance (G_0_ = 2e^2^/h) indicating a tunnel injection process from the electrode into the MWCNT^21^. In this particular case, the conductance directly reflects the MWCNT’s density of states (DOS) allowing scanning tunneling spectroscopy (STS)-like measurements. The measured conductance trace is thus found to be similar to those obtained by STS^22^ and transport measurements^21^ although on much smaller individual CNTs. Two van Hove singularities can be clearly distinguished around ±0.045 eV (marked with ◊) associated with a steep increase of conductance usually characteristic of 1D intersubband quantum transitions^23^. This is remarkable because a clear observation of 1D van Hove singularities associated with the onset of new electronic bands is not expected in large-diameter MWCNTs due to the too small interband spacing as calculated. Conductance oscillations are also observed and marked with ♦ in Fig. 3a. The simulations of the DOS and ballistic conductance (G) for a zigzag TWCNT (same configuration as in Fig. 2b) give a better physical insight (see Fig. 2d) as they reproduce qualitatively the main features measured experimentally in the pseudo-gap regime (Fig. 2b). The opening of the superlattice Dirac point (Fig. 2a) due to a specific 1D Moiré structure (see Fig. 2b) induces also on the DOS profile a set of two ≪super≫ van Hove singularities (i.e. a collection of degenerated van Hove singularities) at slightly different energies. The abrupt increase of conductance at the gap edges could thus be assigned to the simultaneous opening of numerous quantum conductance channels. The superimposed oscillations (♦) also correspond to the opening of new quantum channels of conductance. The residual conductance within the pseudo-gap can be ascribed to the remaining bands present in Γ. Interband coupling is known to be at the origin of this pseudo-gap effect^19^,^20^. In this regime, fractionalization of the conductance carried by each tube was theoretically discussed earlier^19^. Pseudo-gap openings have always remained hard to observe experimentally through conductance measurements because of the weak rotation energy barrier (0.52 meV/atom, c.a. 6 K) predicted for smaller MWCNTs^24^. Blurring of those band structure effects with increasing temperature is expected as they crucially depend on a precise atomic configuration. Although one cannot exclude other chiralities, the presence of highly degenerated new Dirac points appears as a general property of large zigzag MWCNTs (Fig. S2), and the breaking of symmetry induced by tube rotation can induce visible pseudo-gap. A temperature dependence study of the pseudo-gap opening in sample A has been performed and can be found in supplementary information (Fig. S3). The pseudo-gap delimited by the van Hove singularities vanishes slowly around 120 K whereas the oscillations (♦) vanish much faster (around 20 K–30 K). To conclude this section, we stress out the importance of the concept of 1D Moiré effects. It is impossible to describe and understand the presented data (Fig. 2d) with standard models not including intertube interactions and as a consequence superlattice effects.

The opposite chirality limit is the armchair TWCNTs. Theoretical calculations of the band structure of an armchair TWCNT are presented in Fig. 3a,b. For the simulations the (600, 600)@(605, 605)@(610, 610) armchair TWCNT possesses a unit cell containing 7260 carbon atoms and the three tube diameters are 81.4, 82.0, 82.7 nm, respectively. Its electronic band structure is calculated using the same tight-binding approach (Fig. 3a,b and Fig. S4). As for the precedent calculation, Fig. 3a and b differ from the rotation angle of the central tube. Individually, armchair CNTs should present a Dirac cone located exactly at 2/3 along the Γ-X direction of the 1D Brillouin zone. Therefore, without any intertube interactions, the three Dirac cones would superimpose at 2/3 Γ-X. The band structures presented in Fig. 3a,b display a global inversion symmetry with regard to this central position. In contrast to smaller diameter MWCNT, the band structure is highly complex presenting an amount of interpenetrating bands which number strongly depend on the rotation angle of the central tube. Again, superlattice Dirac points and pseudo-gaps can be formed. Degeneracy is less important than for zigzag TWCNTs and Dirac points are opened also within the valence and conduction band due to the bands interpenetration. This is a common property of armchair MWCNTs as it can be observed by increasing the diameter of the tubes (Fig. S5). An example of 1D Moiré pattern corresponding to an armchair TWCNT: (600, 600)@(605, 605)@(610, 610) is illustrated in supplementary information (Fig. S6). For this particular 1D superlattice, the corresponding electronic effects will induce rapidly varying and pronounced features in the DOS and conductance profiles (the energy band separation can be less than 10 meV). Differently to the zigzag TWCNT, these features are still visible far from E = 0 eV. Figure 3c displays the conductance measurement performed at 4.2 K on a second MWCNT (sample B) for which similar features have been revealed. Apart from the absence of pseudo-gap in sample B with respect to sample A, another difference between the two samples is certainly linked to the huge decrease of the contact resistance in sample B. Metal/conducting CNTs interfaces can also give transparent contacts^25^ (few kΩ) comparable to the channel resistance (5 μm long MWCNT for sample B), which seems to be the case of sample B. As the exact chiral indices of the different tubes are not known, the contact resistance could not be unfortunately linked to the nature of contacted shells in this study. A three-probe resistance measurement (and its evolution with temperature) for a large diameter MWCNT can be found in supplementary information (Fig. S7). The calculated DOS and the ballistic conductance are shown in Fig. 3d for the TWCNT configuration corresponding to the case of interpenetrating bands (Fig. 3a). For high contact transparency, the transport measurements should be closer to the calculated ballistic conductance of the TWCNT rather than to the DOS, demonstrating the key role of the contact resistances in the interpretation of the experimental data. By comparison with simulations, different features present in the experimental conductance trace could be assigned to van Hove singularities (marked with ○) and to the zero-bias conductance peak (marked with ●).

Finally, all these 1D Moiré quantum features seem to be stable as they are not blurred even over long transport distances (>1 μm for sample A ; 5 μm for sample B) meaning that the structural quality of the MWCNTs is quite high even if, in Fig. 1a for instance, very slight changes of shape along the MWCNT can be observed. External mechanical strains were surely at play during their insertion in supported devices. In order to better scrutinize the quality of the MWCNTs, Raman spectroscopy has been performed on sample A (see supplementary information, Fig. S8), corroborating the quality of the tube. Considering again the transport properties, the present devices have this important advantage that only the outermost shells are carrying the current thus allowing to consider only a few-shell model that is ≪easier≫ to simulate. The three-monochiral-shell model captures very well the physics of the devices and confirms the presence of 1D Moiré character in the investigated MWCNTs. It is worth noting that some devices displayed also a perfectly V-shaped conductance with a current saturation at high bias as already documented for smaller MWCNTs^26^,^27^ (see Fig. S9 in the supplementary information for a third device – sample C). Simulated conductances presented in Fig. S1 i,j may also exhibit an almost linear variation on the scale [−0.2 eV; +0.2 eV] as in Fig. S9. Such a conductance signature (V-shape) could thus also be compatible with a 1D Moiré pattern produced by monochiral MWCNT. However, as for samples A and B, the chiral indices in sample C are not determined in the experiment and other configurations (i.e. various mixing of chiralities) cannot be unambiguously excluded but are unfortunately inaccessible to simulation with the employed approach and the associated level of accuracy because of the too long size of such systems. A local diffraction technique^28^ probing only the outermost shells would be helpful in this specific case to discriminate. The ideal situation (namely an identical chirality for the three outermost shells) can only happen statistically^28^, although research groups have recently demonstrated the synthesis of pure monochiral MWCNTs^29^,^30^.

In summary, quantum transport properties of 1D Moiré crystals have been investigated both experimentally and theoretically in MWCNTs. By theoretical calculations, specific effects due to superlattices such as the appearance of mini-Dirac points and pseudo-gaps in the electronic band structure have been evidenced. Such effects have been also detected in the low temperature conductance of high diameter MWCNT based solid state devices in different injection regimes. In contrast to twisted graphene bilayers for which the mismatch angle will mainly change the energy position of the mini-Dirac cones^6^, MWCNT 1D Moiré superlattice may induce the opening of these Dirac points thus creating sizeable and robust pseudo-gap electronic structures with possible interesting applications in future MWCNT-based devices. Indeed, in this pseudo-gap regime^19^, the ability to select which shell(s) participate to the conduction is demonstrated to depend on the energy (i.e. the bias voltage or a gate voltage). Such effect can be used, for instance, in spintronic devices to improve the transport of the spin information through the buried shells which are expected to be more protected against spin decoherence than the most outer one being subject to absorption of molecules and atoms onto the C surface and to the presence of the substrate^31^.

## Methods

### A. Tight-binding model

The electronic wave functions of the simulated triple-wall CNTs are expanded on a basis composed of a single atomic orbital per carbon site. The interaction of this π-π* orthogonal tight-binding (TB) model, i.e. the hopping terms 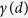, ranges up to a cut-off distance of 2 Å (i.e. it includes only the first-nearest neighbors) in a given CNT : 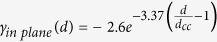 where *d*_*cc*_ = 1.42 Å is the equilibrium interatomic distance. However, the interlayer interaction ranges up to a cut off distance of 3*d*_*cc*_ = 4.26 Å in the direction perpendicular to the nanotube axis: 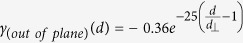 where 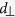 = 3.36 Å is the interlayer distance in graphite^32^.

### B. Devices fabrication

The CVD-grown multiwall carbon nanotubes with large diameter are purchased from MER Corporation. They have been diluted in pure ethanol and then sonicated before dispersion over a plasma-activated Si/SiO_2_ substrate. Individual MWCNTs have been connected thanks to electronic lithography (samples A and C) or optical lithography (sample B) and a subsequent Ni (120 nm)/Au (30 nm) electron-beam evaporation. Evaporation rates (0.05 nm/s) and low pressure around 10^−8^ mBar were kept during the depositions. Electrodes are made thick enough to ensure a complete covering of the nanotube.

### C. Electrical measurement setup

Current-voltage and differential conductance measurements are realized by applying to the device a superimposition of DC and AC signals of low amplitude (1 mV) and at a fix frequency (below 100 Hz). After passing through the sample, the output current is amplified by an I-V converter. A standard homodyne detection allows a direct measurement of the differential conductance while the DC output is measured by a digital voltmeter.

## Additional Information

**How to cite this article**: Bonnet, R. *et al.* Charge transport through one-dimensional Moiré crystals. *Sci. Rep.* **6**, 19701; doi: 10.1038/srep19701 (2016).

## Supplementary Material

Supplementary Information

## Figures and Tables

**Figure 1:**
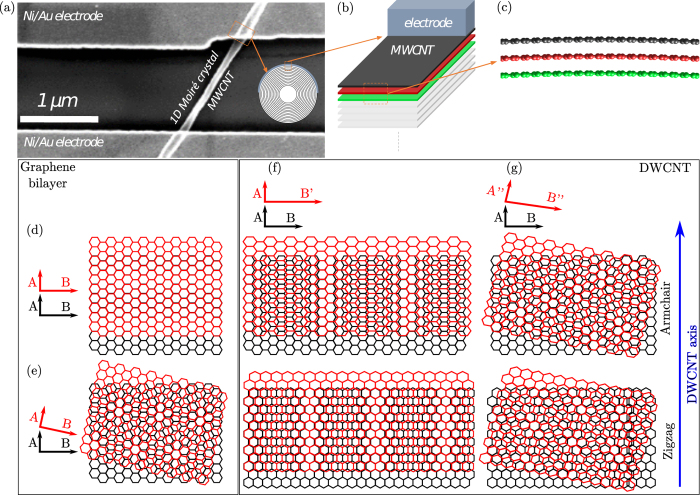
Differences between Moiré patterns in carbon nanotubes and in bilayer graphene. (**a**) SEM image of a device (sample A) composed of a 80 nm diameter MWCNT. Right inset: sketch of the cross-section of the MWCNT close to the contact. (**b**) Enlarged view of the cross section showing the top electrode and highlighting the three most outershells of the MWCNT carrying the current. (**c**) Atomic representation of the three outershells over few C-C distances. (**d–g**) Schematics of Moiré superlattices obtained with two honeycomb lattices either in armchair (upper panels) or zig-zag (lower panels) cases. **A**, **A’**, **A”** (**B**, **B’**, **B”**) represent the lattice vectors of the honeycomb network along (perpendicular) to the nanotube axis. (**d**) AA-stacking in bilayer graphene (no Moiré pattern). (**e**) Twisted bilayer graphene (presence of a 2D Moiré pattern). (**f**) DWCNT with similar tube chiralities revealing a 1D Moiré pattern. One lattice vector is slightly different (**B’** ≠ **B**). (**g**) DWCNT with different tube chiralities revealing a ≪rolled-up≫ 2D Moiré pattern. The lattice vectors are slightly different (**A”** ≠ **A** and **B”** ≠ **B**).

**Figure 2:**
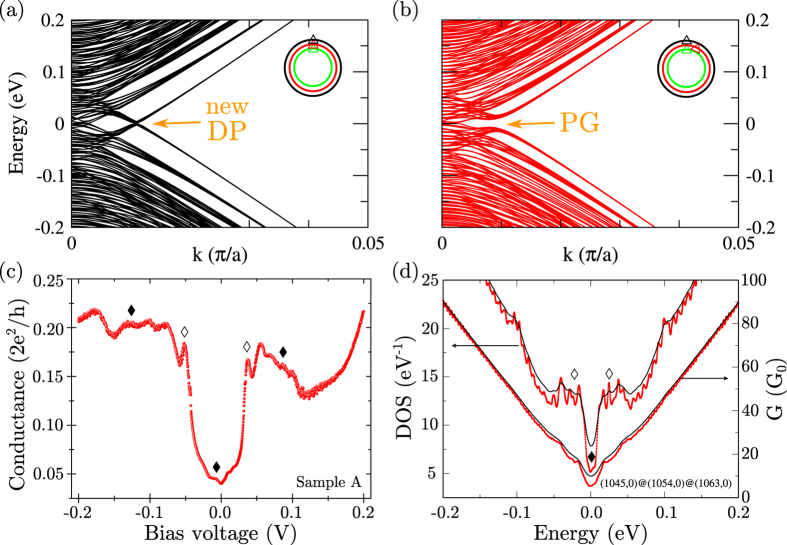
Simulations of electronic properties of a 1D Moiré crystal based on zigzag triple wall carbon nanotubes (TWCNT) and spectroscopy data revealing a pseudo-gap structure on a MWCNT. (**a,b**) Tight-binding band structures of a 80 nm diameter zigzag TWCNT: (1045, 0)@(1054, 0)@(1063, 0). Panels (**a**,**b**) correspond to different rotation angles of the (1054, 0) central tube 
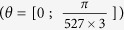
. Superlattice Dirac point (DP) and pseudo-gaps (PG) are indicated by arrows. The Fermi energy (E_F_) is set to zero. (**c**) Conductance measurement performed at 4.2K on sample A with highly resistive contacts. van Hove singularities are marked with ◊ and conductance oscillations due to intershell interactions are marked with ♦. (**d**) Simulated DOS and ballistic conductance of the TWCNT presented in panel (**b**). The red (black) curves are obtained considering a smearing of 2 meV (5 meV). A more complete comparative study of the influence of tube rotation on the band structure, the DOS and on the ballistic conductance can be found in Fig. S1.

**Figure 3:**
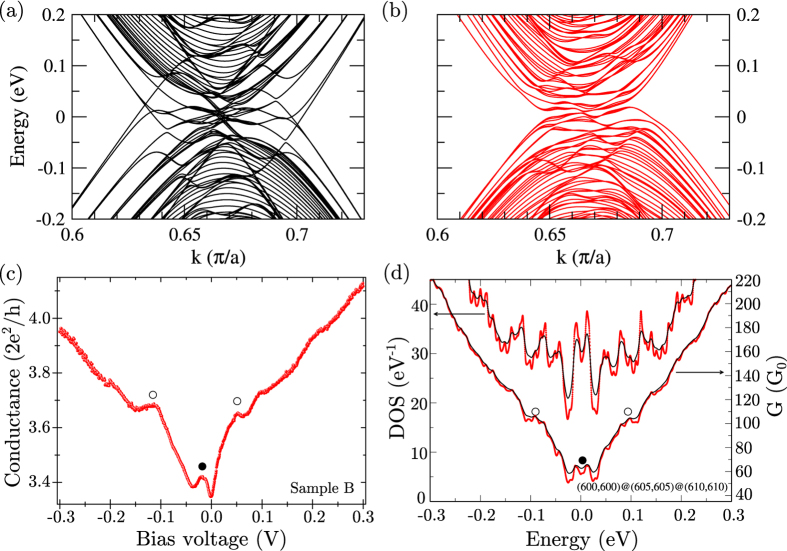
Simulations of electronic properties of a 1D Moiré crystal based on armchair zigzag TWCNT and ballistic conductance mesaurements on a MWCNT. (**a,b**) Tight-binding band structures of a 80 nm diameter armchair TWCNT: (600, 600)@(605, 605)@(610, 610). Panels (**a**,**b**) correspond to different rotation angles of the (605, 605) central tube 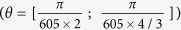. The Fermi energy (E_F_) is set to zero. (**c**) Conductance measurement performed at 4.2K on sample B with transparent contacts. The smoothened van Hove singularities are marked with ○ and the zero-bias conductance peak is marked with ●. (**d**) Simulated DOS and ballistic conductance of the TWCNT presented in panel (**a**). The red (black) curves are obtained considering a smearing of 2 meV (5 meV). A more complete comparative study of the influence of tube rotation on the band structure, the DOS and on the ballistic conductance can be found in Fig. S3.
