# Supplementary material for: Charge transport through one-dimensional Moiré crystals
Source: Sci Rep. 2016 Jan 20;6:19701. doi: 10.1038/srep19701 (PMC4726225; doi:10.1038/srep19701)
Supplement: Supplementary Information [file srep19701-s1.pdf]

Charge transport through one-dimensional Moiré crystals

Roméo Bonnet[1], Aurélien Lherbier[2], Clément Barraud[1], Maria Luisa Della Rocca[1], Philippe Lafarge[1], Jean-Christophe Charlier[2]

[1] Université Paris Diderot, Sorbonne Paris Cité, Laboratoire Matériaux et Phénomènes Quantiques, UMR 7162, 75013 Paris, France

[2] Université catholique de Louvain, Institute of Condensed Matter and Nanosciences, Chemin des étoiles 8, 1348 Louvain-la-Neuve, Belgium

Table of contents

Band structures, densities of states and conductances of triple-wall zigzag nanotubes.
Temperature dependence of the conductance through a 1D Moiré crystal.
Band structures, densities of states and conductances of triple-wall armchair nanotubes.
1D Moiré superlattice.
Intrinsic resistance of a large diameter multiwall carbon nanotube.
Raman characterization of an individual large diameter multiwall carbon nanotube.
Conductance through a “graphene-like” sample.

Band structures, densities of states and conductances of triple-wall zigzag nanotubes.

Tight-binding calculations are presented in Fig. S1, corresponding (from left to right) to the band structures, the densities of states and the ballistic conductances of a zigzag TWCNT (82 nm of diameter) for different orientations of the central tube.

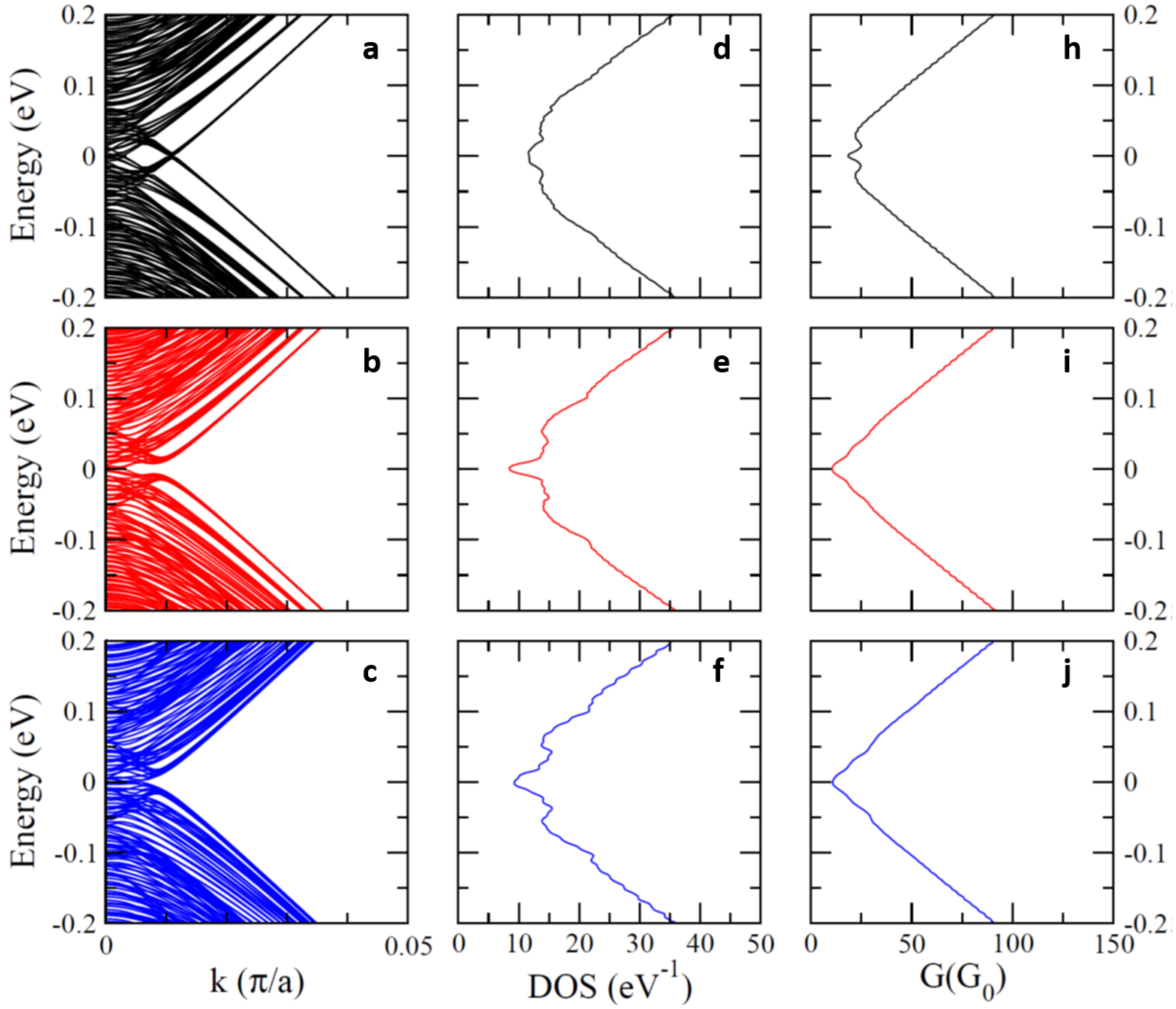

Figure S1. Electronic structure of zigzag triple-wall carbon nanotubes
a-c, Tight-binding band structures of a 82 nm diameter zigzag TWCNT: (1045,0)@(1054,0)@(1063,0). Panels from **a** to **c** correspond to different rotation angles of the (1054,0) central tube ($\theta = \left[0\,;\frac{\pi}{527\times 3};\frac{\pi}{527\times 4}\right]$). **d-f** and **h-j** panels represent the corresponding simulated DOS and ballistic conductances, respectively.

Tight-binding calculations presented in Fig. S2 correspond (from left to right) to the band structures of zigzag TWCNT of various diameters.

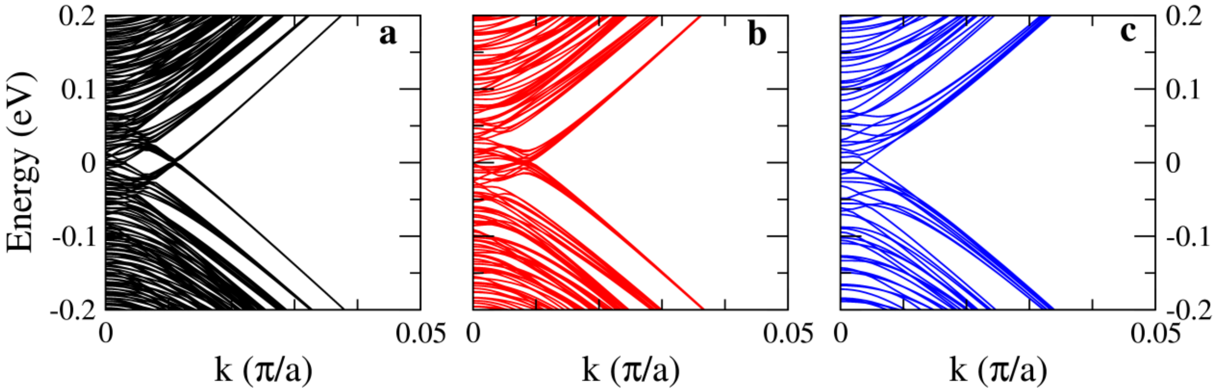

Figure S2. Electronic band structure of various diameter zigzag triple-wall carbon nanotubes
a-c, Tight-binding band structures of zigzag TWCNT of 82 nm, 55 nm, and 28 nm which correspond to (1045,0)@(1054,0)@(1063,0), (697,0)@(706,0)@(715,0), and (349,0)@(358,0)@(367,0) respectively. The central tube rotation angle is $\theta = 0$.

Temperature dependence of the conductance through a 1D Moiré crystal.

The temperature dependence of the conductance for the sample A is presented in Fig. S3. The blurring of the pseudo-gap opening is clearly observed when increasing temperature since this effect is directly correlated to the 1D Moiré superlattice and crucially depends on a specific atomic configuration. The pseudo-gap associated to the van Hove singularities vanishes slowly around 120 K whereas the oscillations vanish much faster (around 20K-30K).

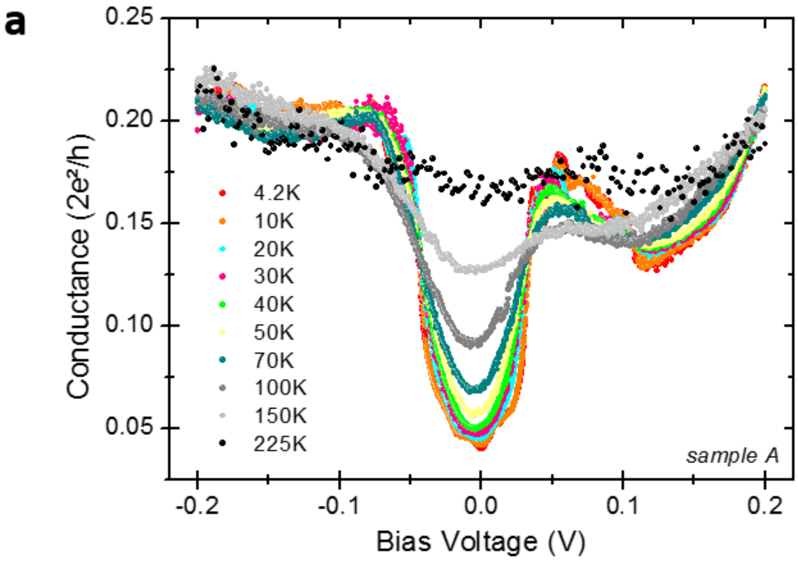

Figure S3. Temperature dependence of the conductance through a 1D Moiré crystal.
a, Temperature dependence of the two-probes conductance (sample A) ranging from 225K to 4,2K.

Band structures, densities of states and conductances of triple-wall armchair nanotubes

Tight-binding calculations are presented, corresponding (from left to right) to the band structures, the densities of states and the ballistic conductances of a armchair TWCNT (Fig. S4) for different orientations of the central tube.

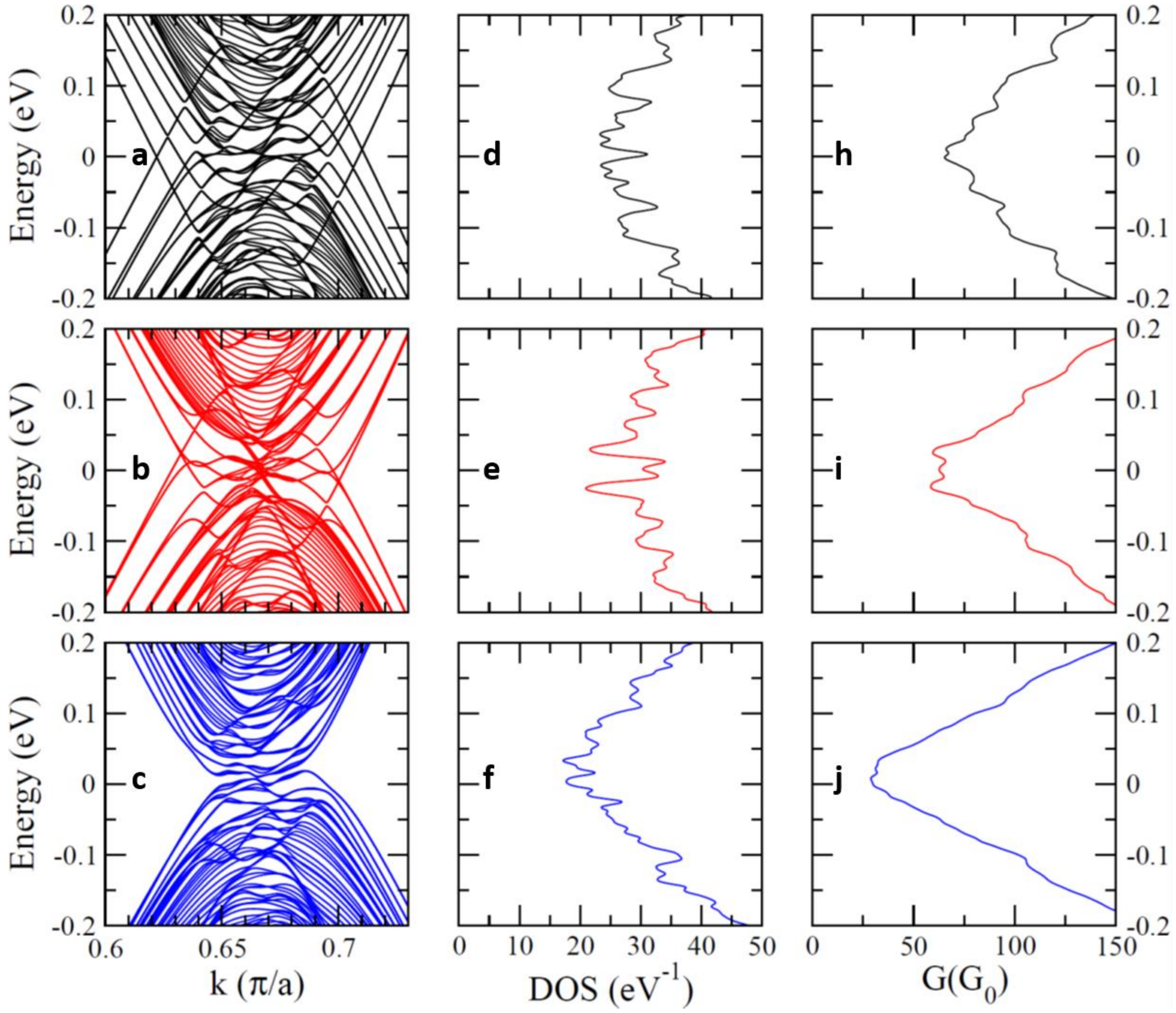

Figure S4. Electronic structure of armchair triple-wall carbon nanotubes

a-c, Tight-binding band structures of a 82 nm diameter armchair TWCNT: (600,600)@(605,605)@(610,610). Panels from **a** to **c** correspond to different rotation angles of the (605,605) central tube ($\theta = \left[0; \frac{\pi}{605\times2}\,; \frac{\pi}{605\times4/3}\right]$). **d-f** and **h-j** panels represent the corresponding simulated DOS and ballistic conductances, respectively.

Tight-binding calculations presented in Fig. S5 correspond (from left to right) to the band structures of armchair TWCNT of various diameters.

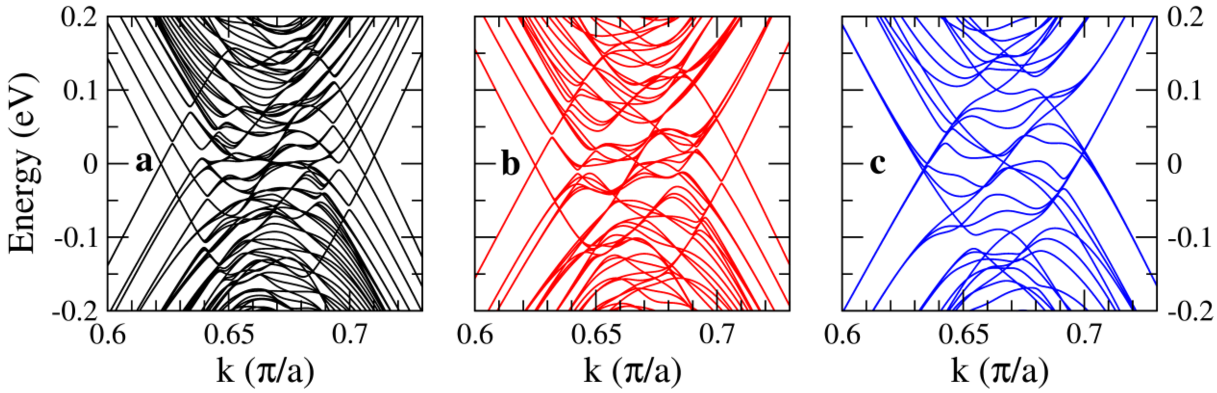

Figure S5. Electronic band structure of various diameter armchair triple-wall carbon nanotubes
a-c, Tight-binding band structures of armchair TWCNT of 82 nm, 55 nm, and 28 nm which correspond to (600,600)@(605,605)@(610,610), (400,400)@(405,405)@(410,410), and (200,200)@(205,205)@(210,210) respectively. The central tube rotation angle is $\theta = 0$.

1D Moiré superlattices.

We present an example to illustrate a 1D Moiré pattern (Fig. S6) corresponding to the simulation shown in Fig. S4.

a

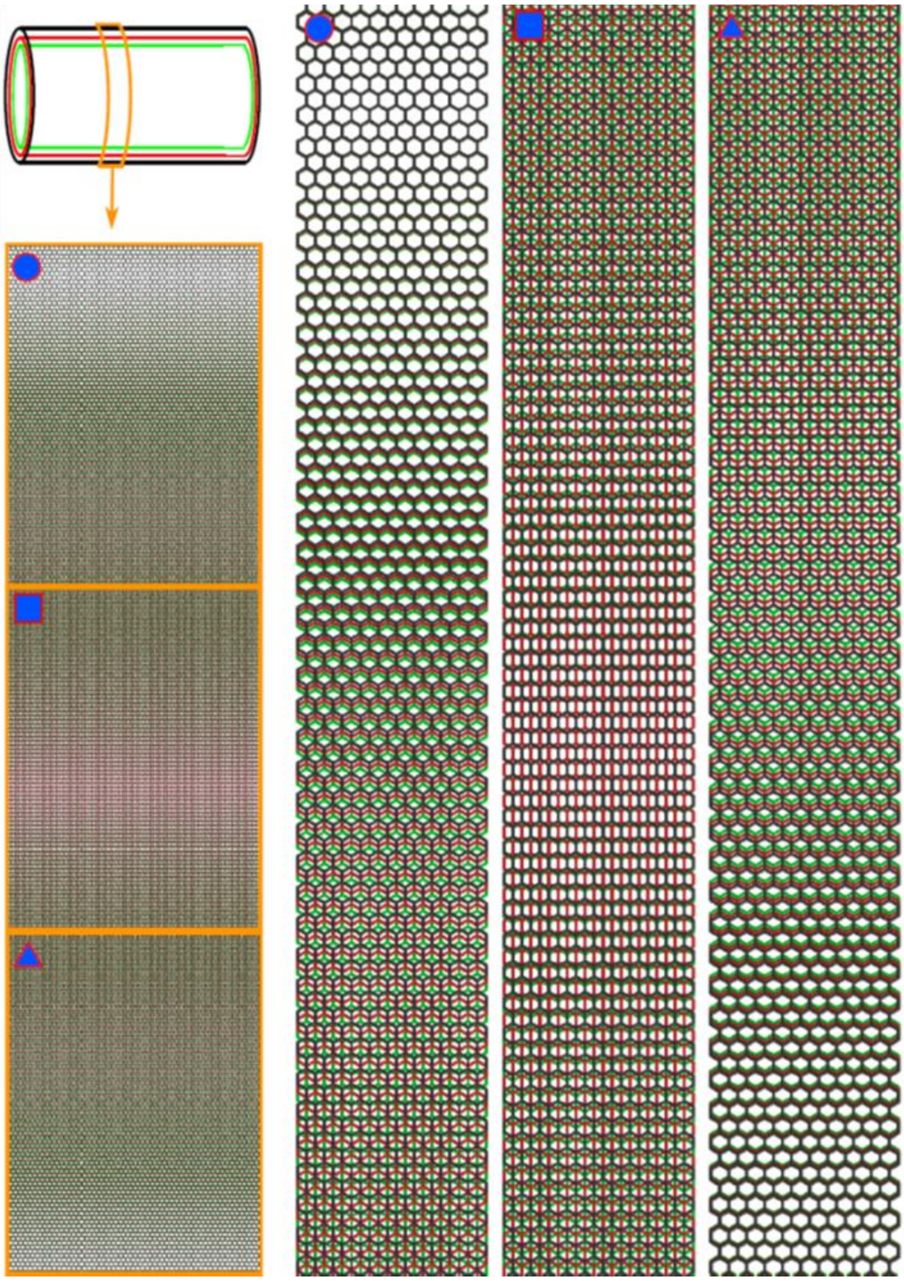

Figure S6. 1D Moiré superlattices.

a, Moiré pattern developped along the circumference of a triple-wall nanotube corresponding to the case presented in Fig. S4b.

Intrinsic resistance of a large diameter multiwall carbon nanotube.

We present in Fig. S7 a temperature dependence of the intrinsic conduction (no contact resistance) of the MWCNT presented in the manuscript in Fig. 3c (sample A). The resistance is extracted from a three-probes measurement as described in the inset. The data are very similar to those reported in Ref. 1.

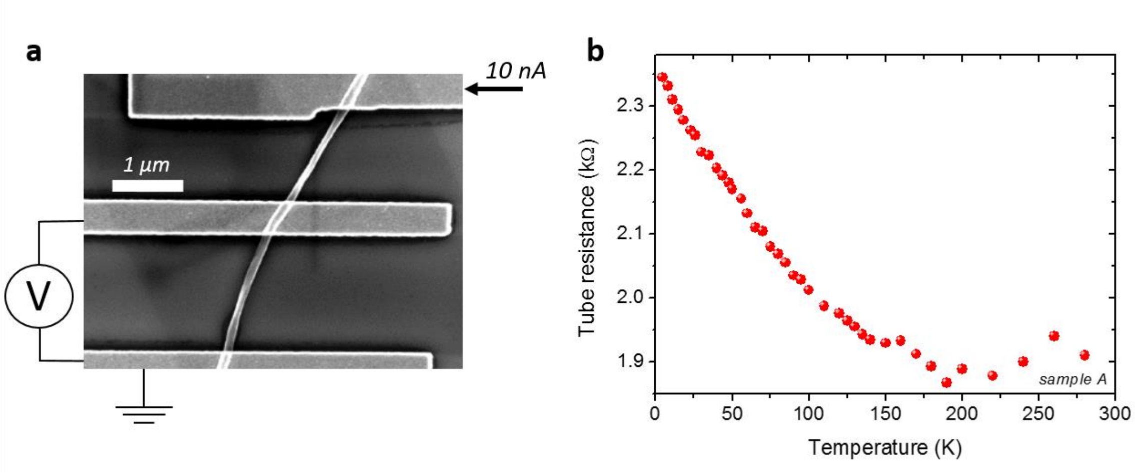

Figure S7: Intrinsic resistance of a large diameter multiwall carbon nanotube.
a, SEM image of the device (sample A). A 10 nA current is injected through the first contact and the potential drop is probed between the two other contacts. **b,** Three-probes resistance measurement as a function of temperature.

Raman characterization of an individual large diameter multiwall carbon nanotube.

We present in Fig. S8 a Raman spectrum (514.5 nm) acquired after processing on the MWCNT presented in the manuscript in Fig. 1a. The probed area is represented by the colored spot indicating also the size of the probed area. Note that this spectroscopy technique is sensitive to the entire structure of the MWCNT[2].

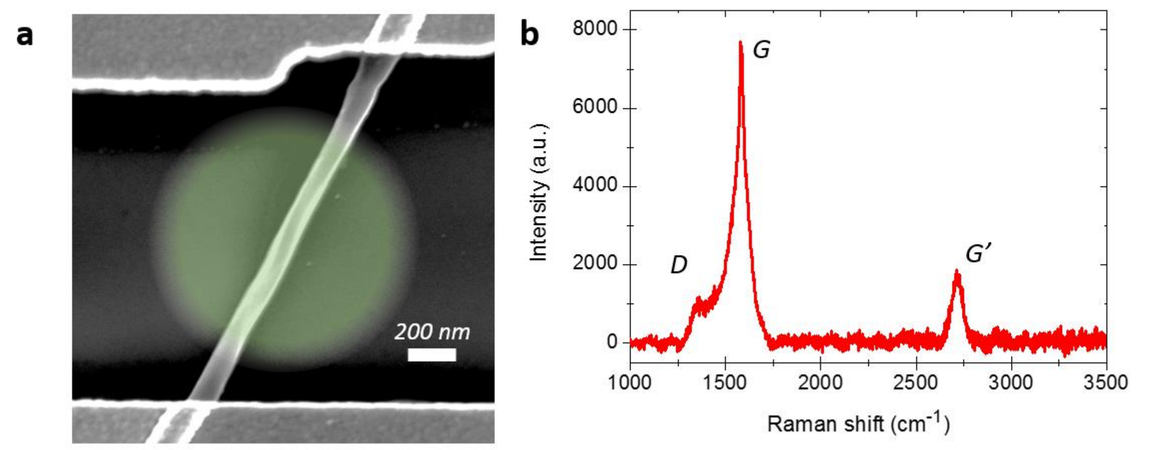

Figure S8: Raman characterization of an individual large diameter multiwall carbon nanotube.
a, SEM image of the probed zone (sample A). The size of the green laser spot is also indicated. **b,** Raman spectra acquired on the multiwall carbon nanotube after processing (power: 1mW, acquisition time: 40s, laser wavelength: 514,5 nm).

Conductance through a "graphene-like" sample.

We present in Fig. S9 a SEM image and a conductance trace obtained at 4.2K with a third device (sample C). At low energy, the variation of the conductance with respect to bias voltage is linear and is similar to data obtained with graphene on graphite[3] and on other MWCNTs[4].

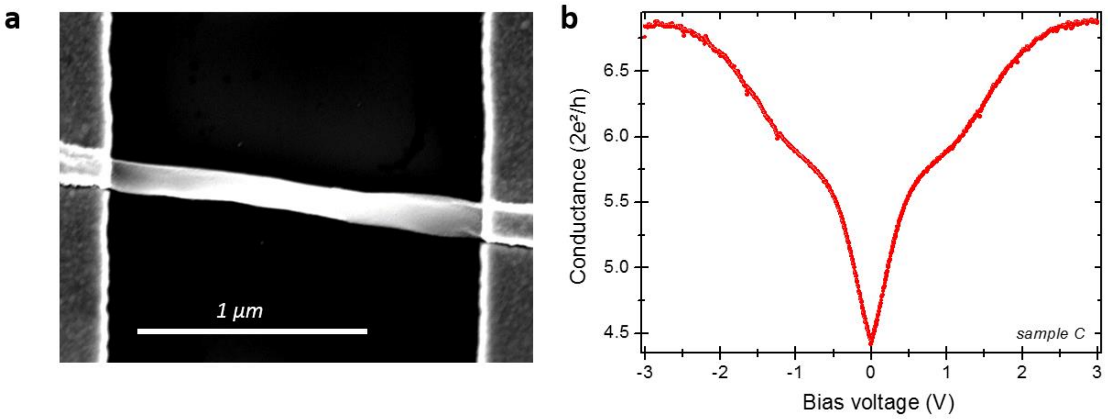

Figure S9: Conductance through a "graphene-like" sample.
a, SEM image of a large diameter multiwall carbon nanotube-based device (sample C). The diameter of tube is 112 nm. **b,** Conductance measured through the device presented in **a** showing a graphene-like electronic structure.

References

1. Schönenberger, C., Bachtold, a., Strunk, C., Salvetat, J. P. & Forró, L. Interference and interaction in multi-wall carbon nanotubes. *Appl. Phys. A Mater. Sci. Process.* **69,** 283–295 (1999).

2. Dresselhaus, M. S., Jorio, A., Hofmann, M., Dresselhaus, G. & Saito, R. Perspectives on carbon nanotubes and graphene Raman spectroscopy. *Nano Lett.* **10,** 751–8 (2010).

3. Li, G., Luican, A. & Andrei, E. Y. Scanning Tunneling Spectroscopy of Graphene on Graphite. *Phys. Rev. Lett.* **102,** 176804 (2009).

4. Liang, Y. X., Li, Q. H. & Wang, T. H. Current saturation in multiwalled carbon nanotubes by large bias. *Appl. Phys. Lett.* **84,** 3379 (2004).
